# *Same/Different* Abstract Concept Learning by Archerfish (*Toxotes chatareus*)

**DOI:** 10.1371/journal.pone.0143401

**Published:** 2015-11-23

**Authors:** Cait Newport, Guy Wallis, Ulrike E. Siebeck

**Affiliations:** 1 School of Biomedical Sciences, University of Queensland, Brisbane, Australia; 2 Department of Zoology, University of Oxford, Oxford, England; 3 Centre for Sensorimotor Performance, School of Human Movement Studies, University of Queensland, Brisbane, Australia; Lund University, SWEDEN

## Abstract

While several phylogenetically diverse species have proved capable of learning abstract concepts, previous attempts to teach fish have been unsuccessful. In this report, the ability of archerfish (*Toxotes chatareus*) to learn the concepts of sameness and difference using a simultaneous two-item discrimination task was tested. Six archerfish were trained to either select a pair of *same* or *different* stimuli which were presented simultaneously. Training consisted of a 2-phase approach. Training phase 1: the symbols in the *same* and *different* pair did not change, thereby allowing the fish to solve the test through direct association. The fish were trained consecutively with four different sets of stimuli to familiarize them with the general procedure before moving on to the next training phase. Training phase 2: six different symbols were used to form the *same* or *different* pairs. After acquisition, *same/different* concept learning was tested by presenting fish with six novel stimuli (transfer test). Five fish successfully completed the first training phase. Only one individual passed the second training phase, however, transfer performance was consistent with chance. This individual was given further training using 60 training exemplars but the individual was unable to reach the training criterion. We hypothesize that archerfish are able to solve a limited version of the *same/different* test by learning the response to each possible stimulus configuration or by developing a series of relatively simple choice contingencies. We conclude that the simultaneous two-item discrimination task we describe cannot be successfully used to test the concepts of *same* and *different* in archerfish. In addition, despite considerable effort training archerfish using several tests and training methods, there is still no evidence that fish can learn an abstract concept-based test.

## Introduction

The ability to form concepts and generalise learned rules is a powerful tool that can allow animals to respond to novel objects or events based on past experience. Natural concepts are also known as categorization, and require the identification of absolute features that are shared by a particular group of items [1]. While items within a category can vary in some respects, they all must share some common features and are therefore item-specific. Abstract concepts, on the other hand, are based on relationships and are therefore not associated with specific stimuli. One commonly studied abstract concept is that of ‘sameness’ and ‘difference’. The particular features that can cause one to designate objects or events as being the ‘same’ or ‘different’ is highly variable and dependent on the context in which the question is asked. As a result, the concept of *sameness* and *difference* can be applied to almost an infinite number of situations.

The formation of abstract concepts requires higher-order reasoning [2] and it has therefore been suggested that only primates are capable of learning this task [3, 4]. Comparative studies examining the ability of non-primate species to learn *same/different* relationships between visual stimuli are limited to relatively few animals such as pigeons [5–13] and to a lesser extent bees [14, 15], dolphins [16], sea lions [17], parrots [18], crows [19], coati [20], harbour seals [21] and fish [22–25]. In these studies, a variety of psychophysical tests were used to determine if subjects could learn the *same/different* relationship for a range of stimuli. The most important source of evidence used to show that subjects could learn the relationship, was the animals ability to accurately solve the test for novel stimuli. Of the species tested, pigeons [11], parrots [18] and harbour seals [21] were shown to be able to successfully apply the learned rules to novel stimuli, however other species, including fish have failed to learn the concept [24, 25].

Fish present an interesting model for comparative cognitive studies as they lack a neocortex, the area of the brain associated with concept learning in primates [26, 27]. Thus far, only three species of fish, goldfish (*Carassius auratus*), cichlids (*Pseudotropheus* sp.) and archerfish (*Toxotes chatareus*), have been tested for abstract concept learning. All three species were tested using a simultaneous matched-to-sample test (sMTS) in which subjects were required to match a sample stimulus to a comparison stimulus. The experiments with cichlids and archerfish both show that they were unable to learn concepts under the particular training conditions employed. The experiments with goldfish were somewhat more promising as in one study the goldfish continued to complete the sMTS task with novel stimuli [23]. However, the same two stimuli were used for all transfer tests allowing the fish to learn how to accurately respond to them [2, 12]. Newport et al. [25] additionally attempted to train archerfish using a procedure based on the concept of oddity. The odd-one-out (OOO) test requires subjects to identify the one stimulus that is different from a group of identical distractors. They found evidence that archerfish could perform a limited version of the OOO test, basing their interpretation on the fact that fish occasionally performed well during training. However, the fish failed to pass the transfer test with novel stimuli. The authors concluded that either archerfish could not learn the concept or that the fish did not fully understand the task on the basis of the training procedures used. In order to actually answer the question of whether fish can apply abstract concepts, we need to first find a testing paradigm that at least allows the fish to pass the training stage. Testing archerfish with an alternative paradigm that has been previously learned by other animals may prove more effective. However, if archerfish are unable to learn another commonly used test, it will add to the accumulating evidence that they are unable to learn abstract concepts, or at least suggest that they can only learn them under very specific training conditions which have not yet been found.

The simultaneous two-item *same/different* discrimination test has been used to determine if pigeons can identify the relationship between two different pairs of symbols [10]. No fish species have yet been presented with this test, however, other animals have been presented with variations of this test including dolphins [16], coati [20] pigeons [10] and bees [14]. In this test, subjects are presented with two pairs of stimuli, one of which has two identical symbols (*same*) and the other that has two different symbols (*different*). Subjects are trained to select one of the two sets. Unlike other possible *same/different* tasks, this particular testing procedure has the advantage that both stimulus pairs are presented simultaneously and can be directly compared within a trial. In this report, we train archerfish to the simultaneous two-item *same/different* test in order to determine the suitability of this paradigm. We followed similar methods to those used by Blaisdell and Cook [10] as they were able to successfully train pigeons to learn this test, but we used black line drawings as stimuli, rather than coloured symbols, as pilot experiments showed archerfish have strong preferences for specific colours which might have distracted them from the task at hand. We assessed the effectiveness of this test based on whether or not the majority, if not all, individuals could pass the training stage and if any individuals applied abstract concept rules in a transfer test with novel stimuli.

## Materials and Methods

### Subjects

Six archerfish were purchased from local suppliers and subjects were maintained as described in Newport et al. [28]. Individual fish had different levels of previous experience with behavioral experiments, however all subjects had at least been pre-trained to spit at stimuli presented on a monitor, following methods described in Newport et al. [28]. Fish 2–5 had all previously participated in multiple concept learning behavioral experiments, while Fish 1 and 6 had only participated in one associative learning experiment.

### General procedure

**Fig 1:**
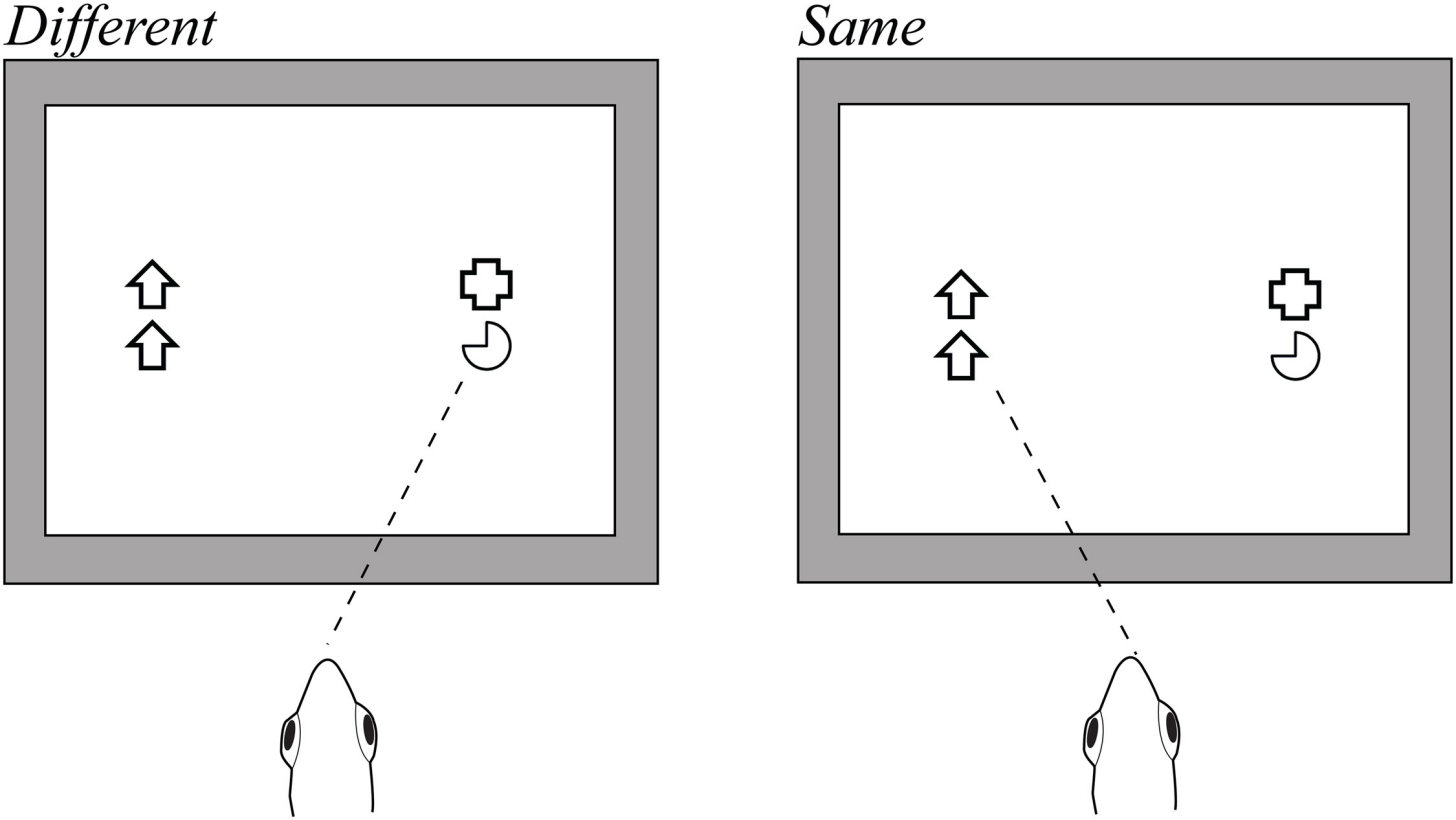
Illustration of the stimulus presentation protocol used in the simultaneous two-item *same/different* discrimination task. Stimuli were a range of line drawings on a white background, presented on a computer monitor suspended directly above the aquarium. In each trial, two pairs of stimuli were presented. In one pair, both shapes were identical (*same*) and in the other pair, both shapes were dissimilar (*different*). Fish 4–6 were trained to select the *same* pair and Fish 1–3 were trained to select the *different* pair. Stimuli not shown to scale.

All experiments were conducted according to the Australian code of practice for the care and use of animals for scientific purposes. The protocol was approved by the Animal Ethics committee of The University of Queensland (AEC Approval number: SBMS/241/12). Stimuli were presented on a computer monitor suspended above the aquaria, as described in Newport et al. [28]. A pair of *same* and *different* stimuli were displayed simultaneously on each trial. One stimulus pair was presented on the left side of the monitor and the other on the right (monitor coordinates: 180 60, 180–60, -180 60, -180–60) with a large gap separating the two pairs (Fig 1). The positions of the *same* and *different* pairs were randomized under the constraint that each pair was never on the same side in more than two consecutive trials. Three fish (Fish 1–3) were trained to select the *different* pair and the other three fish (Fish 4–6) were trained to select the *same* pair. Fish made a selection by spitting a jet of water at the stimuli, henceforth referred to as a ‘hit’. Correct responses (S+) engendered a reward of one food pellet while selection of the incorrect stimulus (S-) terminated the trial without a reward and a 30 second penalty was given before the next trial began. During the initial stages of training, the fish were given the opportunity to continue making selections until they chose correctly in occasional trials. After the fish had made a selection, a squeegee was used to remove water from the Perspex^®^ housing covering the monitor after which the next trial began.

Each session consisted of 30 trials. An individual was considered to have successfully learned the task once the selection of S+ was significantly different from chance in two consecutive sessions. The frequency of S+ and S- selections was tallied per fish for each session and analysed using a binomial test. A S+ selection frequency > 67% in each session is statistically significant (binomial: *P* = 0.0279, *N* = 30 trials).

**Fig 2:**
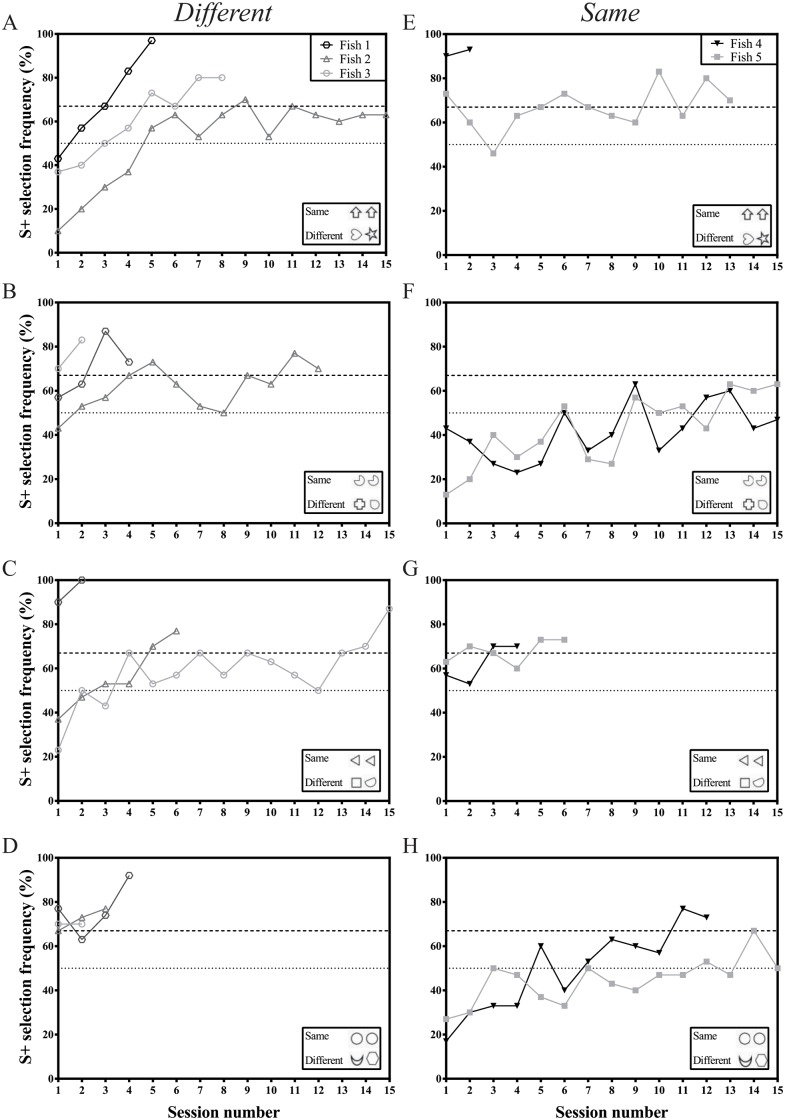
Learning curves of five archerfish conditioned to complete pre-training for the *same/different* task. A-D show results for fish trained to *different* stimuli pairs and E-H show the results for fish trained to *same* stimuli pairs. The dotted line at 50% indicates a S+ selection frequency consistent with chance. The dashed line at 67% indicates the minimum training criterion. Individuals must complete two consecutive sessions above this line, or a maximum of 15 sessions, in order to move on.

A variety of simple line drawings, created using Microsoft PowerPoint and Adobe Photoshop CS5 (Fig 2), were used as stimuli. Each stimulus was approximately 2.5–3 cm in size, depending on its shape. The drawings used have previously been shown to be discriminable by archerfish [28]. Training for this experiment was divided into two phases: 1) Pre-training and 2) Training.

#### Associative pre-training

In pre-training, the same stimuli were used in the *same* and *different* pairs for all trials, allowing the fish to directly associate a particular stimulus pair with a food reward. Once they had learned the task with these stimuli, the fish were then presented with a new set of stimuli. Four successive stages of pre-training were run with four sets of training stimuli. Each fish was trained to a stimulus set until they had successfully reached the training criterion or reached a maximum of 15 sessions. Fish 6 stopped responding after five sessions when presented with Image Set 3 and was therefore excluded from all further experiment and analysis.

The intent of this initial training was to condition the fish to select pairs of stimuli that were the *different* (Fish 1–3) or *same* (Fish 4–6). This is not a true *same/different* test as the fish could have simply memorized the specific stimuli, however, [25] found that this form of initial training can facilitate concept learning. In their experiment, they trained four fish to select a particular stimulus in an odd-one-out task. They then replaced the stimuli and repeated training. When they did this a third time, they found that two of the archerfish immediately selected the correct stimulus and did not require any additional training.

#### Same/different concept training (6 stimuli)

**Fig 3:**
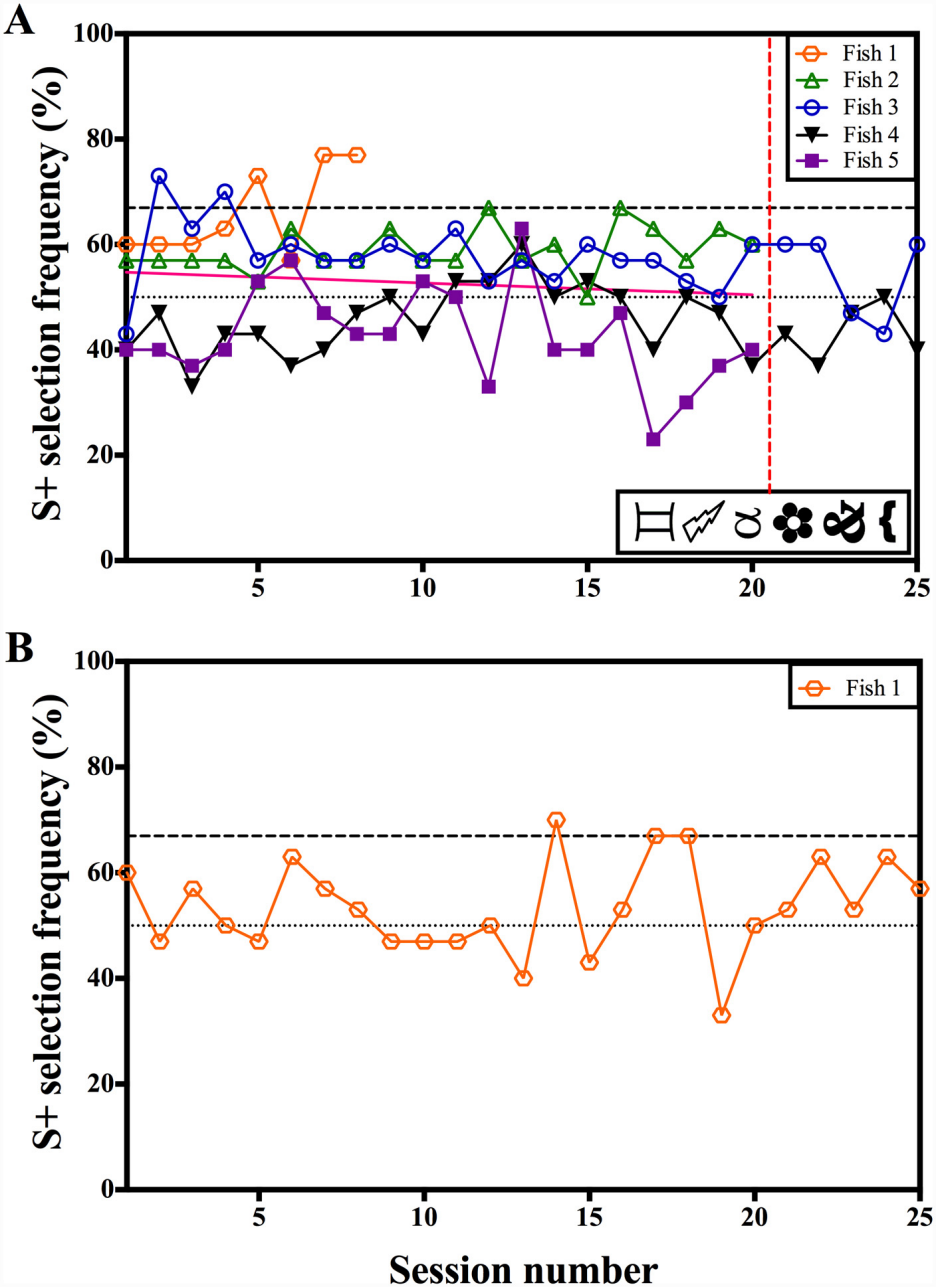
Learning curve of five archerfish conditioned to complete a simultaneous two-item *same/different* discrimination test. The dotted line at 50% indicates a S+ selection frequency consistent with chance. The dashed line at 67% indicates the minimum training criterion: individuals must achieve a S+ selection frequency > 67% in two consecutive sessions within 20 sessions (A) or 25 sessions (B), in order to demonstrated they have learned the test. A) Six training exemplars were used (shown in the figure). The solid pink line is the linear regression fit to the grouped results per session. The vertical dashed red line indicates sessions in which positive punishment was used when fish chose incorrectly. B) 60 training exemplars were used.

Once the archerfish had completed pre-training, they were presented with a true *same/different* task in which the *same* and *different* pairs contained changing stimuli. Six different shapes were used as stimuli (see Fig 3 for shapes) and all could be part of a *same* or *different* pair depending on the trial. Trials within a session were counterbalanced so that all stimuli were in a *same* or *different* pair an equal number of times. A maximum of 20 training sessions were run.

To ensure that at the end of training motivation was still high, two fish (Fish 3 and 4) were given five more training sessions in which a penalty was given in the form of a distasteful food reward. Denatonium benzoate is an extremely bitter, nontoxic compound used to prevent nail biting in humans, chewing in dogs and as an animal repellent. In addition, it has been previously used in experiments to teach fish to avoid unpalatable prey [29]. Bitter tasting food pellets were made by soaking them in a solution of 2 milligrams (mg) of denatonium benzoate per millilitre (mL) of ethanol and allowed to dry completely. This concentration has previously been shown to be effective in causing wrasses to avoid a food item [29]. When archerfish chose the incorrect stimulus during training, they were fed one bitter food pellet. It was expected that the fish would spit out the food and a small hand-net would be used to immediately remove the unwanted pellet from the aquarium.

#### Same/different concept transfer test (6 stimuli)

Once a fish had successfully passed the training, the fish were given a transfer test to determine if they could apply the *same/different* rules to novel stimuli. Six new shapes replaced the previous stimuli. Each session consisted of 30 trials and fish were tested for two sessions. Each of the six stimuli was part of a *same* pair once every five trials and twice as part of a *different* pair (once on the left and once on the right side of the pair).

#### Same/different concept training (60 stimuli)

One fish (Fish 1) completed the *same/different* training but failed the transfer test (see Results section for details). Pigeons are more likely to learn a concept when a large number of training stimuli are used [11] therefore, the small number of training exemplars (six) may have affected the rules learned by the fish. To test this, a further 25 training sessions were given to Fish 1 with 60 new training exemplars. Each stimulus was used once in a *same* pair and twice in a *different* pair every 60 trials.

#### Shape discrimination and general learning control

**Fig 4:**
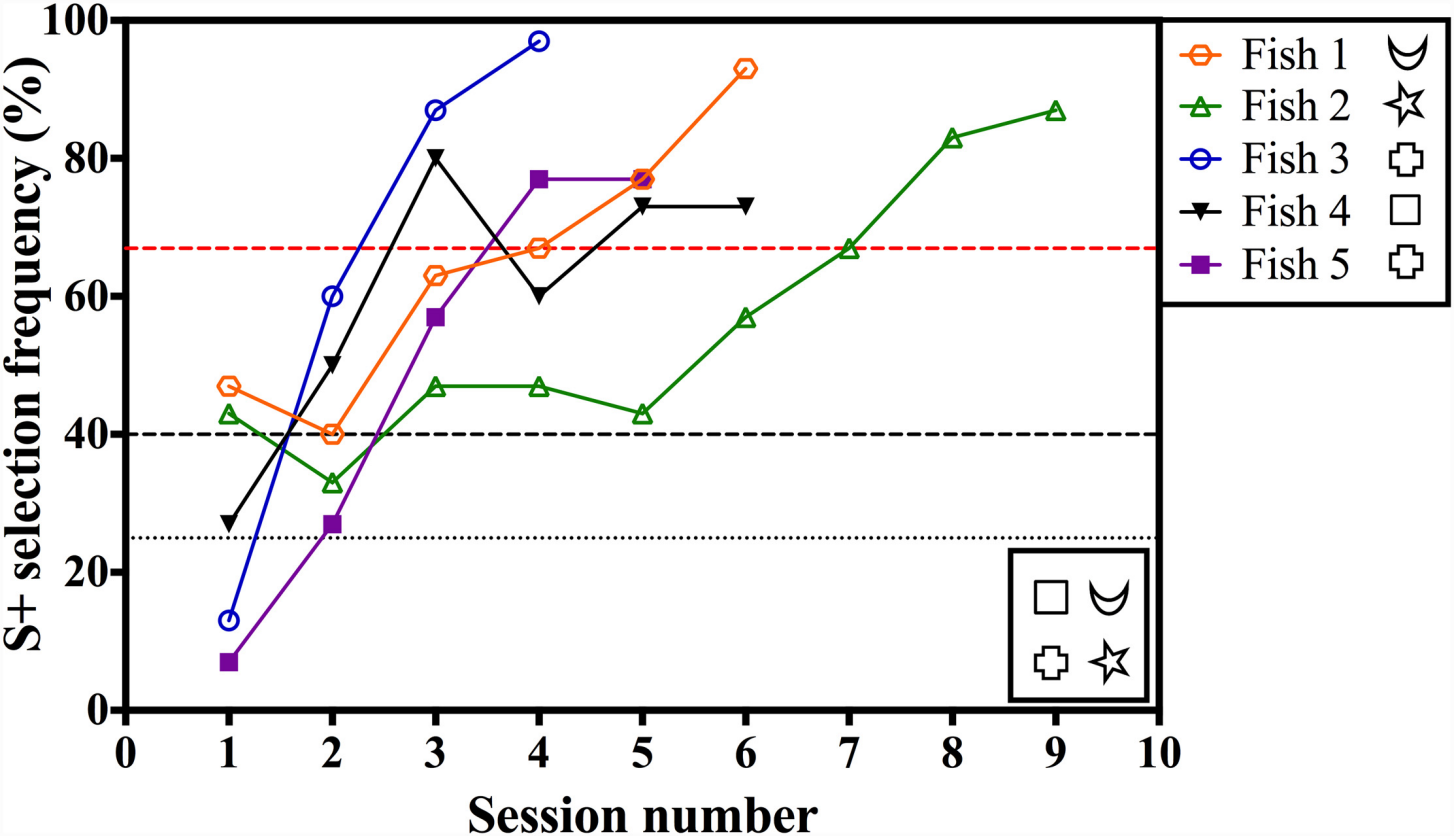
Learning curves of five archerfish when trained to discriminate four shapes (shown in the figure) using a four-alternative forced-choice test. The black dotted line indicates a S+ selection frequency consistent with chance. The black dashed line indicates a S+ selection frequency significantly different from chance. The red dashed line marks the S+ selection frequency stipulated as the training criterion.

A final test was run as a control to ensure that each fish was still motivated to learn and that the types of stimuli used in training could be discriminated by the individuals in this experiment. Fish were presented with a 4-alternative forced-choice (4-AFC) test using stimuli from the associative pre-training test (one shape from each step). These same shapes had successfully been used in a similar 4-AFC by Newport et al. [28]. Each individual fish was trained to select a different shape (see Fig 4 for shapes), except two fish that were trained to select the same shape (Fish 1: crescent; Fish 2: star; Fish 3: cross; Fish 4: square; Fish 5: cross). There were four stimulus display positions on the monitor (monitor coordinates: -200 150, 200 150, -200–150, and 200–150) and the positions of all stimuli were randomized with the constraint that S+ was never in the same position in consecutive trials. Trials were recorded as either being ‘correct’ (selection of S+) or ‘incorrect’ (selection of any of the three S-). Sessions consisted of 30 trials and were run until each fish had reached an S+ selection frequency > 67% but with a maximum of 15 sessions. A S+ selection frequency of > 40% is significantly different from chance (binomial: *P* = 0.029, *N* = 30 trials), however the training criterion was set to match that of the two-choice *same/different* test.

## Results

### Associative pre-training

Four stimulus sets were used in pre-training. When presented with Image Set 1, four of the five remaining fish reached the training criterion within 15 sessions (Fish 1: 5 sessions; Fish 3: 8 sessions; Fish 4: 2 sessions; Fish 5: 13 sessions) but Fish 2 did not (Fig 2A and 2E). When trained to Image Set 2 (Fig 2B and 2F), both fish trained to *same* (Fish 4 and 5) did not learn the task within 15 sessions but all fish trained to *different* did (Fish 1: 4 sessions; Fish 2: 12 sessions; Fish 3: 2 sessions). Only Fish 3 was unable to learn the task when presented with Image Set 3 (Fish 1: 2 sessions; Fish 2: 6 sessions; Fish 4: 4 sessions; Fish 5: 6 sessions) (Fig 2C and 2G). Fish 5 was again unable to learn the task within 15 sessions when trained to Image Set 4 but all other fish completed the task (Fish 1: 4 sessions; Fish 2: 3 sessions; Fish 3: 2 sessions; Fish 4: 12 sessions) (Fig 2D and 2H).

### *Same/different* concept training and transfer test (6 stimuli)

Five archerfish moved on to the *same/different* training (Fig 3A). After 20 training sessions, only Fish 1, which was trained to select *different*, met the training criterion. Fish 3 also reached a statistically significant S+ selection frequency in two sessions however these sessions were non-consecutive. Given that the probability of reaching our learning criteria by chance in a particular sessions is *P* = 0.0279 (*N* = 30 trials), we would expect about 4 sessions to be statistically significant due to chance for all five fish (0.0279*30*5 = 4.455). We observed five sessions where the selection frequency was significantly different from chance. In order to reduce variability in the results due to daily variation in individual fish performance, the mean S+ selection frequency for all fish was grouped per session. A linear regression was then used to fit a line to these results to determine if there was any improvement over time. While the slope of this line was slightly negative (-0.2247 ± 0.1324), it was not significantly different from zero (R^2^ = 0.138, *F*(1,18) = 2.880, *P* = 0.1069).

Fish 3 and 4 were given a further 5 training sessions where a bitter tasting food pellet was given when fish chose incorrectly. This positive punishment did not produce any increase in performance accuracy and neither fish reached the training criterion during this period.

Fish 1 reached the training criterion therefore two transfer test sessions were given in which the original six training stimuli were replaced for six novel ones. An S+ selection frequency of 53% was reached in both sessions. This S+ selection frequency was not significantly different from chance (binomial: *P* = 0.135, *N* = 30 trials).

### *Same/different* concept training (60 stimuli)

Only Fish 1 proceeded to the final training stage in which 60 training exemplars were used (Fig 3B). Fish 1 was given 25 training sessions but was unable to meet the training criterion within that time. Performance reached above statistical significance in only one session, which is expected due to chance (0.0279*30*1 = 0.837).

### Shape discrimination and general learning control

A 4-AFC test was run as a control to test whether the fish were still motivated to learn (Fig 4). All fish reached the training criterion within nine sessions (Fish 1: 6 sessions; Fish 2: 9 sessions; Fish 3: 4 sessions; Fish 4: 6 sessions; Fish 5: 5 sessions).

## Discussion

We tested whether archerfish can learn the abstract concept of *same/different* using a training procedure previously applied to pigeons [10]. Pre-training was used to familiarize the archerfish with the general procedure of the *same/different* test. In this phase the stimuli used were not changed until the fish reached the learning criterion, allowing the fish to solve the test using direct association with the stimuli. All fish were capable of learning the pre-training. When presented with the same procedure but where the stimuli in the *same* and *different* pairs were variable, all archerfish but one failed to pass the training criterion even after significant training (600 trials) and the addition of positive punishment (150 additional trials). Fish 1 did learn to complete the test but failed to transfer to a novel set of stimuli. When trained with a larger number of exemplars, Fish 1 failed to reach the training criterion. As a final step, the fish were trained to a 4-alternative forced choice test. All fish tested were able to learn this within 4–9 sessions showing that despite the poor performance during the *same/different* task, all fish were capable of learning a discrimination test to a high degree of accuracy (maximum S+ selection frequency for all fish was between 73–97%). Because five out of the six fish tested were unable to even pass the initial training criterion, we conclude that the simultaneous two-item *same/different* discrimination test is an ineffective method to test concept learning in archerfish. While it is possible that an individual could eventually learn the task using the method described, the majority of the fish would not. As a result, most fish that take part in the experiment would have to be excluded from testing, making this a laborious and inefficient process.

The results of our experiment are negative; however, it is impossible to know whether that is because archerfish are entirely incapable of concept learning, or if the training process did not effectively communicate the task. In this case, it seems unlikely that the failure of the archerfish to learn the task was simply due to minor procedural details such as number of trials per session, feeding schedule or stimulus size. A range of fish species have been successfully trained by multiple research groups, all following different procedures, showing that fish can learn associative-based tasks despite trivial differences in training procedure. The stimuli used in this experiment have previously been shown to be distinguishable by archerfish [25, 28] and the success in the pre-training show that archerfish can learn the general procedure of the test. It is possible that given more time the archerfish may have eventually learned the task. In fact, Bisazza et al. [30] found that when conditioning guppies, extensive training time can improve performance. However, guppies improved after 120 trials while we saw no improvement in archerfish performance after 600 trials. Newport et al. [25] noted that archerfish seemed to stop attempting to learn new decision rules after about 10–20 training sessions (300–600 trials) and instead resorted to making selections based on a particular stimulus or stimulus position.

While minor procedural details are unlikely to have stopped the archerfish from learning, we believe the general procedure of the simultaneous two-item *same/different* discrimination test is not appropriate for archerfish. This test is a feasible training method as it has been successfully employed with pigeons [10]; however, this is the first time it has been used with fish. We hypothesized that this test may be more successful than the matched-to-sample (MTS) or odd-one-out (OOO) tests attempted by Newport et al. [25] because, unlike in the OOO and MTS procedures, this test allows the subjects to see both the *same* and *different* conditions simultaneously. However, even pigeons experience some difficulty learning this task when presented with only two items [10]. Presenting a greater number of *same* and *different* stimuli within a session, such as the arrays described by Young et al. [31], or using a larger set of stimuli during training, such as those used by Katz and Wright [11], may serve to better highlight the relationship between stimuli. Young et al. [31] observed the greatest transfer test performance when pigeons were presented with 12–16 stimuli in a trial and Katz and Wright [11] observed the highest transfer accuracy when using a training stimuli set of 1,024 different images. Newport et al. [25] had previously attempted to train archerfish to complete an OOO test using non-repeating stimuli in a pilot study, but found little evidence of improvement throughout training despite the large number of training exemplars. Perhaps a combination of more stimuli presented in each trial and larger stimuli training sets would be the best approach for future experiments.

Alternatively, the stimuli themselves, rather than the number, may have prevented the fish from identifying the relationship between stimuli. Although archerfish are capable of discriminating the stimuli used in this study [28], they may find it easier to identify relationships between stimuli that are more natural to them, such as other fish, insects or leaves. In this study, we chose stimuli that are likely unfamiliar to archerfish in order to avoid preferences or biases for familiar objects which may affect our results. In pilot studies, we found that archerfish have strong biases to some colours. For example, while archerfish will spit at a variety of coloured dots without hesitation, we found that many individuals would not spit at a red dot regardless of how much time they were given. In addition, the fish would stay in the bottom corner of their tank rather than swimming relaxed at the top of the water. Interestingly, when two red dots were presented, many of these fish were willing to participate in experiments again. As we do not know the full extent of biases archerfish have, we felt using ecologically plausible stimuli may unwittingly affect our results. Ben-Tov et al. [32] successfully used moving bars as stimuli and found these elicited a strong response in archerfish. However, we do not think moving stimuli can be incorporated into a *same/different* test such as ours. It is feasible that one group of stimuli could all move in the same direction (*same*) and the other group could move in different directions (*different*) but finding stimuli to be used in the transfer test is problematic as we do not feel that changing the movement direction of the stimuli would be adequately novel to be considered a true transfer test. In addition, it would be difficult to determine whether the archerfish were responding to the relationship between the moving stimuli or being affected by pop-out effects (which is what Ben-Tov et al. [32] used the moving stimuli to test). Many psychophysical tests have been applied to test whether animals can learn abstract concepts and it is possible that one of these variations will be successful; however, so far archerfish have failed to learn four of the more common psychophysical tests (OOO, simultaneous MTS, delayed MTS and the *same/different*).

Positive punishment, in the form of a bitter food reward, was used to increase the incentive for fish to make a correct choice. This had no significant effect on the accuracy of the two fish tested. The positive punishment may have had no effect because the archerfish were simply unable to learn the task or because it may also not have been the most effective form of positive punishment. Although denatonium benzoate has previously been used to train fish to avoid particular prey based on visual cues [29], in our experiments, the archerfish almost always ingested the pellet. Prior to testing, the fish were given the bitter pellets and typically took the pellets in their mouths but quickly spit them out again, at which point the pellets were fished out of the aquarium. During testing, the fish stopped spitting the pellets out or if they did, they would quickly eat them again before the pellet could be removed from the aquarium. This may indicate that the concentration used was too low or that denatonium benzoate is not distasteful enough to archerfish. The goldfish trained by Zerbolio and Royalty [23] were faced with a much more severe outcome for incorrect choices as shocks were used as reinforcement. Future experiments may have more success if a more severe form of negative reinforcement is used.

Not all fish were unable to learn our test as Fish 1 did reach our training criterion. However, this individual did not appear to solve the test using the concept of *same/different* as performance was consistent with chance when novel stimuli replaced the learned ones. It is likely that the fish instead learned to select S+ using a strategy based on item-specific associations. For example, stimulus configuration learning or a multiple-rule model could be used to solve the test [12, 33]. If the fish had learned separate responses to each configuration of the six stimuli, it would require that the fish learn 60 unique stimulus combinations. If the fish considered the same stimulus in a different position to be different, then the number of unique combinations increases to 240. Although this method would require an impressive degree of memorization, it has the benefit of being very accurate. Alternatively, multiple *if-then* contingency rules might have been employed. This requires less rote memorization but accuracy is dependent on how well the learned rules fit the actual data. Fish 1 may have applied *if-then* rules, rather than the configural model, as it appeared to make selections based on the presence of a particular stimulus. For example, Fish 1 chose the ‘lightning bolt’ symbol in every trial it appeared and almost always avoided the ‘flower’ and ‘parentheses’ symbol. Both of these strategies are item-specific which explains why the S+ selection accuracy of Fish 1 decreased when presented with novel stimuli. The increase in training exemplars to 60 symbols should make it significantly more difficult for Fish 1 to apply item-specific associative rules. Ultimately Fish 1 did not show the same level of S+ selection accuracy when more training exemplars were used. Fish 1 was the only individual that achieved the training criterion but it is not the only fish to have employed an item-specific strategy. Many of the other fish appeared to solve the test by only selecting a particular side or stimulus. Because of the way the trials were balanced, this solution only allowed the fish to achieve an accuracy consistent with chance. These preferences often changed from session to session (e.g. in one session the fish preferred any stimuli on the left side and in the next session they preferred any stimuli on the right) likely reflecting the fact that the fish were attempting to find a strategy that increased their likelihood of receiving a reward.

Based on the results from this report as well as those by Newport et al. [25], it appears that archerfish are unlikely to learn concepts when trained using the more common psychophysical tests, if at all. The evidence from other fish species is equally ambiguous. Gierszewski et al. [24] tested whether cichlids could learn a simultaneous matched-to-sample (sMTS) procedure using two shapes as training stimuli and found that none of the fish could learn the test. Goldfish were shown to successfully learn a sMTS test; however, only two stimuli were used during training and no transfer trials were run [22]. Based on this experiment it is impossible to say whether the fish had learned to solve the test by learning the concept of matching or whether, like the archerfish, the goldfish had simply learned item-specific associations. A second experiment with goldfish also showed that they could learn a sMTS test using two coloured lights as training stimuli [23]. Transfer tests were run with novel stimuli and the performance within the first block of trials was equal to baseline trials. However, only two stimuli were used as novel stimuli in transfer trials and a single block of trials consisted of the results of three sessions (40 trials each) for all four goldfish. As a result, the first block of transfer trials consisted of the results of 480 trials with only two stimulus configurations. The fish had ample time to learn associative-based rules within the first block especially considering that positive punishment was used, which may increase the rate of learning. To avoid the potentially confounding factor of stimulus repetition, transfer testing should have ideally been done with a larger number of novel stimuli or the statistical analysis of the results should have accounted for repeated measures [12]. Based on the five studies conducted to date with fish (including this report), there is still no direct evidence that fish can learn abstract concepts. However, this is a very small number of studies with even fewer species of fish. Future experiments using different testing methods and different species of fish would add considerably to our knowledge of the cognitive abilities of fish.

Archerfish have an impressive ability to rapidly learn item-specific associations even compared to other fish species [28]. It is possible that abstract concept solutions are simply not relevant to archerfish and that associative strategies provide the most accurate and reliable results for archerfish and are therefore preferred. Archerfish are generalist feeders that make rapid decisions when downing aerial prey [34–37] and they encounter many prey items that are similar in appearance. Following prey selection strategies that are too abstract may mean that suitable prey are unnecessarily avoided. For example, if archerfish encounter an aposematic insect that is green, learning the rule that all green species are unpalatable may mean that they miss out on green but non-toxic insects. Alternatively, there may be a greater advantage for archerfish to apply natural concepts rather than abstract ones as other species have fish have been shown to have some degree of categorization ability [38]. However, this is speculative. It is equally possible that all fish in general simply lack the neural structures for the level of processing complexity required for abstract reasoning. Determining whether other species of fish can learn this task would provide important information about the role that ecology or neural structures plays in this form of learning.

The results of this experiment, as well as those by Newport et al. [25], suggest that archerfish do not learn abstract concepts using the particular training methods described. While it is important to know what animals can do, it is equally important to understand their limitations. In this case, the inability of archerfish to learn several concept-based tests raises several interesting questions. If archerfish are truly incapable of learning the concepts associated with the tests, then we might ask if this is a general trend across all fish species or whether there is something specific that limits archerfish. If all fish species are incapable, then what is it that is different between fish, insects and birds that enable only the bees and birds to learn abstract concepts? Unlike birds and bees, when archerfish are tested using the same procedures, most individuals are not even getting past the training stage, let alone passing the transfer tests. In all of the tests presented to the archerfish thus far, the training stage can be solved using associative-based strategies. Archerfish can learn to implement complex decision rules [28] even though they may require significant memory resources. So why are the archerfish described here often failing to even pass the training stage? More experiments focused on answering these specific questions are required if we are to understand the limits of fish cognition and whether or not they truly have the capacity for abstract concept learning.
